# Barriers to breastfeeding and targeted breastfeeding social transfer programs in São Paulo, Brazil: a qualitative study

**DOI:** 10.1186/s13006-026-00865-2

**Published:** 2026-06-19

**Authors:** Stephanie Khoury, Ana Carolina Onofre, Barbara Sartorio, Kelly Coca, Maira Luchini Costa, Najmeh Karimian-Marnani, Sonja Merten, Alexandra Brentani, Jordyn Wallenborn

**Affiliations:** 1 University of Basel, Basel, Switzerland; 2 Department of Epidemiology and Public Health, Swiss Tropical and Public Health Institute, Allschwil, Switzerland; 3 Faculdade de Medicina da Universidade de São Paulo (FMUSP), São Paulo, Brazil; 4 Child Development Center (CEDI), Department of Pediatrics, Faculdade de Medicina da Universidade de São Paulo (FMUSP), São Paulo, Brazil; 5 Paulista School of Nursing, Universidade Federal de São Paulo (UNIFESP), São Paulo, Brazil

**Keywords:** Exclusive breastfeeding, Barriers, Social transfer programs, Qualitative research, Brazil

## Abstract

**Background:**

Exclusive breastfeeding (EBF) is recommended for the first six months of life, yet only 48% of infants under six months are exclusively breastfed worldwide. Breastfeeding rates vary significantly across regions, countries, and even within cities. Brazil reports national rates of EBF of approximately 50%; however, there are notable in-country socioeconomic disparities, where EBF prevalence is lower in low-income communities compared to the wealthier communities. This study aims to: 1) investigate barriers to breastfeeding by exploring perceptions and experiences of breastfeeding in low-income communities in São Paulo, Brazil, and 2) evaluate the perceptions of a targeted social transfer program to improve EBF prevalence at six months.

**Methods:**

We conducted a semi-structured, exploratory, qualitative study in low-income communities in São Paulo, Brazil using focus group discussions (FGD) and key informant interviews (KII). Three FGDs were conducted, two with mothers and one with fathers (n = 14), alongside five KIIs with key stakeholders, including a nurse, neonatologist, social worker, and representatives from the Brazilian Ministries of Health and Social Development (n = 5), with a total sample of 19 participants. An inductive thematic analysis approach was used to develop the codebook and identify recurrent themes across transcripts.

**Results:**

Despite the overall positive outlook on breastfeeding, FGDs and KIIs revealed several challenges, including economic, health system, and sociocultural barriers that hinder both initiation and continuation of breastfeeding. A targeted social transfer program was identified as a potential mechanism to address these barriers and support breastfeeding outcomes.

**Conclusions:**

The identified barriers highlight the complexity of breastfeeding in low-income settings. Strengthening the existing breastfeeding social transfer program, Auxílio Nutriz, may serve as a supportive mechanism to address identified barriers and support breastfeeding practices. Understanding these challenges is a critical step toward developing coordinated, cross-sectoral interventions that support sustained breastfeeding practices. These findings have important implications for public health policy and program design, providing an evidence base for targeted interventions aimed at reducing inequities in breastfeeding outcomes among vulnerable populations.

**Trial registration:**

The RCT was registered on ClinicalTrial.gov on December 6, 2023 (NCT06157697); https://clinicaltrials.gov/study/NCT06157697?term=STEBB%26rank=1.

**Supplementary information:**

The online version contains supplementary material available at 10.1186/s13006-026-00865-2.

## Background

Breastfeeding is essential for child survival and health, providing a safe, natural, nutritious, and sustainable nourishment [1]. Beyond reducing infectious morbidity and mortality, breastfeeding is associated with improved cognitive development, fewer dental malocclusions, and a lower risk of obesity and chronic diseases in life. For mothers, breastfeeding confers protective effects against breast and ovarian cancers, type II diabetes, and supports birth spacing. Improving breastfeeding rates globally could prevent an estimated 823,000 child deaths and 20,000 breast cancer deaths annually [2, 3].

Exclusive breastfeeding (EBF) is defined as providing infants with only breast milk without any other liquids or solids except for medically indicated supplements. Based on systematic reviews from 2002 and 2012, the World Health Organization (WHO) recommends that infants be exclusively breastfed for the first six months of life, with continued breastfeeding alongside complementary feeding up to two years [4, 5]. Despite these long-standing guidelines, in 2023, only 48% of infants under 6 months of age were exclusively breastfed worldwide [1].

EBF rates vary by region and country, depending on environmental, socioeconomic, and demographic factors, particularly in areas with significant inequities. In Brazil, persistent inequalities play a role in shaping breastfeeding practices, particularly among mothers in low-income communities [6]. Over the last 40 years, Brazil has made substantial progress in breastfeeding promotion through a series of comprehensive and innovative public health policies, including expanded maternity leave legislation, public awareness campaigns, and initiatives incentivizing primary health care professionals to support breastfeeding mothers [7–10]. Additionally, the Baby Friendly Hospital Initiative, which aims to promote breastfeeding immediately after birth, led to the establishment of the world’s largest network of human milk banks and the regulation of infant food marketing [11, 12].

Despite these advances, recent data indicate a slight decline in breastfeeding indicators since 2006, suggesting that existing strategies are insufficient to meet the Ministry of Health’s (MoH) target of 70% EBF by 2030 [8, 13]. In 2019, Brazil’s national EBF rate was at 46%, above the Latin America and the Caribbean (LAC) regional average of 43% [14]. However, disparities exist within the country and within cities, with higher EBF rates typically found in high-income communities compared to low-income areas [14]. A study in São Paulo found an average EBF rate of 39% with significant variations in the prevalence rate by residential area: ranging from 33% to 62% [15]. These findings highlight the significant variation in EBF prevalence across different socioeconomic communities and underscore the need for a better understanding of existing barriers, as well as more targeted initiatives to promote EBF.

A potential strategy to improve EBF rates and address breastfeeding disparities is the implementation of social transfer programs targeting mothers of low socioeconomic status. Such programs provide financial or material support to encourage breastfeeding while alleviating poverty. Social transfer programs have been successfully implemented in the past; however, the majority have focused on improving health and educational outcomes [16–18]. Brazil’s Bolsa Família program, a national conditional social transfer program launched by the Ministry of Social Development and Fight Against Hunger (MoSD) in 2003, targets poor households with children under 18 years of age [19]. Families receive a social transfer of at least 600 Brazilian Reais, contingent upon meeting conditions such as going to routine health visits and ensuring school attendance for children [20]. This program has been successful in reducing poverty rates and improving health and education outcomes among children and adolescents [21].

Given the success of Bolsa Família and the association between lower family income and reduced breastfeeding duration, integrating a social transfer directed at supporting breastfeeding into the program could help address EBF barriers and support breastfeeding practices [22]. In June 2023, the Brazilian government launched the Benefício Variável Familiar, Auxílio Nutriz, one of the Bolsa Família complementary unconditional social transfer of 50 Brazilian Reais to support nursing mothers in the first six months postpartum [23]. This program is the only nationally implemented program that provides financial support for breastfeeding mothers and is not systematically linked to structured breastfeeding education or awareness campaigns [23].

As a newly implemented program, Auxílio Nutriz has not yet been evaluated, and evidence on how it is perceived by beneficiary households and key stakeholders remains limited. At the time of data collection (April to June 2024), the program had been in place for approximately one year, allowing for early insights into stakeholders and community perceptions during its initial implementation phase. A qualitative study is therefore valuable to identify context-specific perceptions and experiences related to breastfeeding, understand breastfeeding challenges, and explore how the current social transfer program is interpreted, accepted, and experienced in practice. This study aims to 1) investigate barriers to breastfeeding by exploring perceptions and experiences of breastfeeding in low-income communities in São Paulo, Brazil, and 2) evaluate the perceptions of a targeted social transfer program to improve EBF prevalence at six months. In this study, the term “social transfer program” is used to refer broadly to interventions providing financial or material support to mothers.

## Methods

We conducted a semi-structured, exploratory, qualitative study, in São Paulo, Brazil. The study included three focus group discussions (FGDs) with a total of fifteen participants involving both mothers and fathers, as well as five key informant interviews (KIIs) with relevant stakeholders.

All FGDs and KIIs were conducted between April and June 2024. Two FGDs were conducted with mothers (four and six participants) and one with fathers (four participants). The FGDs were conducted separately for mothers and fathers to facilitate open discussions and to minimize potential power dynamics or social desirability bias in mixed-gender groups. The KIIs included interviews with MoH and MoSD representatives, two with healthcare workers, and one with a social worker.

### Eligibility criteria

For the FGDs, all mothers and fathers: 1) had at least one child under two years of age, 2) lived in the Butantã Jaguaré district in São Paulo, 3) did not have a medical, intellectual, or psychological disability, and 4) agreed to participate and sign an informed consent form. Participation in social transfer programs, such as Bolsa Família, was not an inclusion criterion. Information on participation in such programs was only collected during FGDs with mothers, as these programs are typically directed toward them as primary caregivers. Participation in Auxílio Nutriz was not specifically assessed, as participants were not always aware of their enrollment and may only become aware once the benefit is received at six months postpartum.

For the KIIs, the ministry representatives, social worker, and healthcare workers: 1) did not have a medical, intellectual, or psychological disability, and 2) agreed to participate and sign an informed consent form.

### Study recruitment

The initial KII participants were identified by the project’s co-principal investigator based on their professional roles and relevance to the study objectives. Additional participants were approached through referrals from interviewed stakeholders. Due to the participants’ time constraints, all KIIs were conducted via zoom.

Snowball sampling was used to recruit FGD participants to facilitate recruitment community-based recruitment and build trust. The project coordinator, who works in the study district of Butantã Jaguaré, identified initial participants who were then invited to participate and asked to nominate others. While this approach may have influenced participant diversity, efforts were made to include both mothers and fathers with varied household characteristics (Table 2). FGDs took place at the University of São Paulo Medical School. To support participation, transportation along with drinks and snacks were provided. No financial incentives were offered.

While larger FGDs were initially planned, participant availability and last-minute cancellations resulted in smaller group sizes. In practice, these smaller groups facilitated more in-depth and comfortable discussions, allowing participants greater opportunity to share their experiences.

### Procedure

Semi-structured interview guides for KIIs and FGDs were developed based on study objectives, relevant literature, and the research team’s experience in maternal and child health. Interview guides were reviewed and pilot-tested by the co-principal investigators to refine question clarity and flow (Supplementary Material). Participants were asked about their perceptions and experiences related to breastfeeding and infant feeding practices, including views on breastfeeding and formula use within their communities. Discussions explored influences on feeding decisions, such as partners, family members, and healthcare providers, as well as broader contextual factors including work responsibilities, caregiving dynamics, and household decision-making. Participants were also invited to reflect on potential breastfeeding support interventions, including social transfer programs, with a focus on their perceived feasibility, acceptability, and potential impact.

KIIs were conducted by the co-principal investigator, and FGDs were facilitated by a trained research assistant. All interviews were conducted in Portuguese, audio-recorded on a secure device, and lasted approximately 45 to 60 minutes (KIIs) and 100 to 120 minutes (FGDs). Thematic saturation was assessed iteratively throughout data collection and analysis by the research team. After each FGD and KII, preliminary themes were discussed and compared to previously identified codes. Saturation was considered achieved when no new themes or subthemes emerged in the final interviews, and when additional data did not contribute further conceptual insights to the existing thematic framework. While additional FGDs were initially considered, data collection was concluded once thematic saturation was reached, indicating that the number of groups was sufficient to address the research objectives.

Ethical approval for this study was obtained from the Ethics Commission of Northwestern and Central Switzerland (EKNZ; 2024-00015) and the Brazilian National Commission for Ethics in Research (CONEP; 7.643.369). All participants provided both written and oral informed consent prior to participation and were informed of their right to withdraw at any time. All data were de-identified and stored securely with access strictly to the research team.

### Data analysis

The audio recordings were transcribed in Portuguese using the sonix.ai and reviewed for accuracy by the Brazilian research team. Transcripts were translated into English using DeepL Translator and cross-checked by both the Swiss and Brazilian teams for quality control.

An inductive thematic approach was used to develop a codebook and identify recurrent themes that emerged from both FGD and KII transcripts. Multiple codes could be assigned to a single quote when more than one relevant concept was represented. Two researchers independently coded the data in both Portuguese and English using Dedoose (v.9.2.22). Both coders had prior training and experience in qualitative research. The codebook was refined iteratively throughout the coding process, with bi-weekly meetings to compare coding and resolve discrepancies. If differences persisted, a third member of the research team reviewed and validated the coding.

Translation accuracy was ensured through dual-language coding, independent review by bilingual team members, and cross-checking of key excerpts against the original Portuguese transcripts during analysis discussions. Inter-rater reliability was assessed using a pooled Cohen’s Kappa coefficient. This process was conducted for both the KIIs and FGDs.

### Research reflexivity

The research team included members with backgrounds in public health, social work, and clinical practice, based in both Switzerland and Brazil. The involvement of local researchers familiar with the study context facilitated participant trust and open dialogue. However, the researchers’ affiliation with academic institutions may have introduced perceived power dynamics. To mitigate this, interviewers emphasized neutrality, encouraged open and non-judgmental discussion, and clarified that there were no right or wrong answers. Reflexive discussions were conducted throughout data collection and analysis to consider how researchers’ perspectives and positionality may have influenced interpretation.

## Results

Demographic characteristics of participants that completed the KIIs and FGDs are displayed in Tables 1 and 2. The mean age of the FGD participants was 33 years and the majority were unemployed (43%) or worked in informal jobs (36%). Among the FGDs with mothers, 40% were Bolsa Família recipients. All KIIs were completed by women working in formal jobs with an average age of 50 years.

**Table 1:** Characteristics of the key informant interview (KII) participants

Characteristics	KII 1	KII 2	KII 3	KII 4	KII 5
Gender	Female	Female	Female	Female	Female
Age	43	47	72	43	45
Number of children	2	2	2	2	2
Race	White	White	White	White	White
Occupation	MoH Representative	Social Worker	Neonatologist	MoSD Representative	Nurse

KII = Key Informant Interview

MoH = Ministry of Health

MoSD = Ministry of Social Development and Fight against Hunger

Gender and race were self-identified by participants. Racial categories follow the Brazilian Institute of Geography and Statistics (IBGE) classifications

**Table 2:** Characteristics of the focus group discussion (FGD) participants

Characteristics n (%) or mean (SD)	FGD 1 (*n* = 4)	FGD 2 (*n* = 6)	FGD 3 (*n* = 4)
Gender	Females	Females	Males
Age^1^	35 (9.6)	29.1 (4.4)	37.8 (10.8)
Number of children^1^	1.3 (0.5)	1.6 (0.8)	3.3 (1.5)
Race	White: 3 (75%) Black: 1 (25%)	White: 5 (83%) Black: 1 (17%)	White: 1 (25%) Black: 3 (75%)
Bolsa Família recipients	Yes: 2 (50%) No: 2 (50%)	Yes: 2 (33%) No: 4 (67%)	-
Occupation	Informal: 2 (50%) Unemployed: 2 (50%)	Formal: 1 (17%) Informal: 1 (17%) Unemployed: 4 (67%)	Formal: 2 (50%) Informal: 2 (50%)

^1^ The variable is continuous – the values are shown as a mean, with the standard deviation in brackets

FGD = Focus Group Discussion

SD = Standard Deviation

Gender and race were self-identified by participants. Racial categories follow the Brazilian Institute of Geography and Statistics (IBGE) classifications

### Barriers to breastfeeding

Participants described breastfeeding barriers across three interrelated domains: economic, health system, and sociocultural. Although presented separately, these domains were often experienced as overlapping and mutually reinforcing. Within each domain, subthemes were identified to capture the specific mechanisms through which barriers influenced mothers’ ability to initiate and sustain breastfeeding.

Interpretations of these barriers varied by participant role. Mothers and fathers primarily emphasized the realities of caregiving and their experiences with breastfeeding. Healthcare professionals and policy stakeholders, while often framing breastfeeding challenges within broader institutional and structural contexts, also drew on their own personal experiences as mothers. Together, these differing viewpoints highlight how breastfeeding barriers are simultaneously experienced at the household level and shaped by broader systemic factors.

#### Economic barriers

Participants reported economic barriers as directly shaping their ability to breastfeed, particularly through the need to return to work shortly after childbirth. Financial insecurity, informal employment, and insufficient maternity protection were identified as key influences on feeding decisions.

##### Precarious employment and inadequate maternity protection

Unstable employment conditions and limited maternity protections emerged as central constraints on mothers’ ability to breastfeed. Mothers working in informal sectors reported lacking legal protections, paid leave, or workplace accommodation for breastfeeding, often requiring them to return to work shortly after delivery.

A MoH representative emphasized the vulnerability of mothers employed in informal work:

*Our big challenge is the informal worker, who has no employment rights, and who sometimes returns to work a week or two after the baby is born … otherwise there’s no money in the house. (KII 1, 43 years old)*

Mothers highlighted how maternity leave policies were misaligned with breastfeeding recommendations:

*How do you say that women have to breastfeed exclusively for up to six months, if most workers’ maternity leave ends at four [months] … So I think we’d have to think about guaranteeing maternity leave of at least six months and a reduction in the workload up to a year … Because the math doesn’t add up. (FGD 1, 40 years old)*

Community participants similarly recognized the economic rationale for breastfeeding, with some fathers framing it as both a natural and financially necessary choice:

*We go by nature, we go by instinct. Like when I had my first daughter, I was in a very bad financial situation. So the smartest, most obvious solution was to keep breastfeeding, because you don’t spend anything. (FGD 3, 40 years old)*

These accounts highlight how work and income constraints are key influences on breastfeeding continuation. Both mothers and policy stakeholders described EBF as difficult to sustain when employment demands and financial pressures limited the time and conditions necessary for continued breastfeeding.

#### Health system barriers

Health system barriers emerged as a distinct domain in which institutional practices and professional guidance shaped mothers’ breastfeeding decisions. Participants identified inadequate guidance and inappropriate promotion of formula by healthcare providers as major health system influences on their feeding decisions.

##### Inadequate guidance from healthcare providers

Mothers reported receiving inconsistent or harmful recommendations from healthcare professionals, introducing uncertainty and undermining confidence in their breastfeeding decisions.

One mother recalled receiving advice that directly contradicted established breastfeeding recommendations:

*I went to one pediatrician with my newborn … and he told me to give him breastmilk every three hours, and to make a liter of chamomile or lemon tea, put a spoonful of sugar in it and give it to my son seven days old. (FGD 1, 32 years old)*

Another mother described a difficult hospital experience with a nurse during her first latching attempt:

*One of the nurses tried to put the nipple in the baby’s mouth, but she squeezed very, very, very hard. I couldn’t stand the pain and she didn’t have the patience …. But in the end she gave up on me … she left me there and didn’t give any advice. (FGD 2, 36 years old)*

These accounts reflect how harmful advice and insufficient hands-on support shaped mothers’ early breastfeeding experiences

##### Inappropriate formula promotion by healthcare providers

Inappropriate formula promotion by healthcare providers shaped mothers’ perceptions of feeding options, even with the Baby Friendly Initiative and the International Code of Marketing of Breast-milk Substitutes in place [12, 24, 25].

One mother described doctors as actively discouraging breastfeeding in favor of formula:

*There are a lot of doctors who still recommend formula instead of breast milk … There are doctors who discourage breastfeeding, in order to put in formula. (FGD 1, 40 years old)*

A MoH representative linked this to gaps in professional training:

*We fail a lot in training our professionals … if you have professionals who don’t understand the importance of breastfeeding … they will easily be harassed by the industry and fall for the industry’s talk. (KII 1, 43 years old)*

While mothers noted how provider recommendations directly shaped their feeding decisions, stakeholder’s account identify systemic training failures that leave professionals vulnerable to industry influence.

#### Sociocultural barriers

Sociocultural barriers to breastfeeding emerged within households and broader community and public environments. Participants identified gender norms and perceived and embodied realities of breastfeeding as key influences on feeding decisions.

##### Gender norms

Gender norms shaped breastfeeding practices across multiple dimensions, from rigid division of household labor to partner authority over feeding decisions and sexualization and appearance-related expectations. Participants consistently noted how these norms operated as immediate constraints on mothers’ ability to initiate and sustain breastfeeding.

###### Rigid division of household labor

Participants reported limited household support structures in which mothers were expected to maintain domestic responsibilities alongside infant care, while fathers were culturally positioned as uninvolved in feeding decisions.

A MOH representative working with the Bolsa Família program emphasized that breastfeeding depended on redistributing household labor:

*When we talk about encouraging breastfeeding within the family, we’re talking about someone having to do the work that woman did before the child was born … Because if that woman has to do that and breastfeed, it’s breastfeeding that’s going to be negatively affected … (KII 4, 43 years old)*

Fathers acknowledged how cultural norms discouraged shared caregiving responsibilities, framing breastfeeding support as outside their role:

*Most [men] are going to be indifferent about [breastfeeding] … We’ve never heard at school that men have to help … It’s simply the woman’s obligation and that’s it. (FGD 3, 51 years old)*

###### Partner authority over infant feeding decisions

Beyond the division of labor, gender norms also shaped power dynamics around infant feeding decisions. One mother described how a partner’s control over his wife’s body directly determined her feeding choice:

*I knew [someone] who didn’t want his wife to breastfeed his daughter, because he said that breasts weren’t for children. Understand? He didn’t want her to. And then … she didn’t breastfeed her daughter because her husband didn’t want her to. (FGD 1, 32 years old)*

Participants also described how partner jealousy could translate into pressure on mothers to stop breastfeeding:

*It’s just that there are some men who are jealous of their wives. Like, she’s breastfeeding, and her breasts are exposed here … the husband becomes jealous. (FGD 2, 24 years old)*

These accounts illustrate how breastfeeding decisions were negotiated within relationships where fathers’ attitudes could constrain mothers’ choices.

###### Sexualization and appearance-related expectations

Gender norms further manifested through concerns about sexualization and bodily appearances following pregnancy and childbirth, often tied to fear of partner rejection.

One mother described how appearance concerns led someone she knew to avoid breastfeeding entirely:

*I know … people who chose not to breastfeed … because she thought her breasts would sag and she didn’t want to lose the aesthetics … She didn’t want to lose her looks, she had silicone … then she got pregnant and didn’t want to. (FGD 1, 32 years old)*

Healthcare professionals similarly observed that body image concerns affected mothers’ confidence and were frequently tied to partner dynamics:

*I think this aesthetic still exists. Some mothers don’t want to breastfeed because they say their breasts will sag and their husband will reject them. And then there’s all this work of convincing and guiding them so that they don’t worry about it. (KII 3, 72 years old)*

Mothers also described experiences of harassment and unwanted male attention when breastfeeding in public, which further compounded feelings of discomfort and insecurity:

*I felt insecure [breastfeeding in public] … there are other men standing around, just watching … you don’t stop being abused on the bus, even visually. You feel abused, watched, like you’re a piece of steak in a man’s hand. (FGD 2, 25 years old)*

Participants noted how appearance-related pressures and experiences of public harassment shaped their comfort and decisions around breastfeeding

##### Perceived and embodied realities of breastfeeding

Despite widespread recognition of breastfeeding’s importance, participants described perceived and embodied realities of breastfeeding shaped by stigmatization, concerns about breast milk adequacy, and breastfeeding-related pain.

###### Stigmatization of breastfeeding

Mothers reported experiencing judgment and social pressure around breastfeeding, particularly when they encountered difficulties breastfeeding.

One mother described the tension between cultural messaging and everyday realities:

*It’s because we consider breastfeeding to be very important … it helps with immunity and everything, right? And then you see everyone saying: “Oh, breastfeeding is important, you have to give milk to your child, you have to do it” And then you’re extremely judged if you can’t or don’t, it’s a sad thing. (FGD 1, 32 years old)*

Participants also noted a contradiction between societal promotion of breastfeeding and the absence of public spaces that would allow mothers to breastfeed comfortably and feel safe doing so:

*We talk about breastfeeding, about the importance, but we don’t have a society where we feel at ease and comfortable breastfeeding. We don’t have places to breastfeed. We go out on the street but there’s no place where you can [breastfeed] … You need to hide in the public bathroom. (FGD 1, 23 years old)*

Participants reported stigmatization from two directions—being judged for not breastfeeding, and facing discomfort and harassment when attempting to breastfeed in public spaces

###### Concerns about breast milk adequacy

Doubts about milk adequacy frequently led families to consider formula supplementation. Fathers’ interpretations were often based on perceptions of infant behavior, leading them to view breast milk as insufficient:

*I think breast milk ends up no longer supplying the child’s hunger … the breast is no longer satisfying. (FGD 3, 25 years old)*

Mothers also described how perceived low milk supply led some women to stop breastfeeding despite wanting to continue:

*I know some women haven’t been able to breastfeed. They suffered terribly because they thought it was important, but they couldn’t do it. There was no milk. (FGD 1, 40 years old)*

A neonatologist noted that social comparison with formula-fed infants further reinforced these perceptions:

*Mothers believing their milk is weak … they think that the baby isn’t putting on enough weight. That their milk isn’t good … they compare it to the neighbor’s child, who is on formula, and the neighbor’s baby is putting on more weight than hers. (KII 3, 72 years old)*

Notably, concerns about milk adequacy were echoed across mothers, fathers, and healthcare professionals, suggesting these doubts were embedded within the broader feeding culture.

###### Breastfeeding-related pain

Pain during early breastfeeding emerged as a shared concern among mothers in the community, with participants reporting how experiencing or hearing about pain led to fear about initiating and continuing breastfeeding.

A mother explained:

*It’s really painful at the beginning … it feels like you’re tearing your breast … then they start to pull, then the breast rips and more blood comes out with the milk than the milk itself … I think I see a lot of people wanting to give formula to avoid the pain. (FGD 2, 40 years old)*

Another mother similarly noted how pain was widely cited within her social network as a reason for early breastfeeding cessation:

*The few that I’ve spoken to … give up because of the pain and suffering of breastfeeding at first. Many mothers have told me that they have given up. (FGD 2, 32 years old)*

Breastfeeding-related pain was frequently cited as a reason for introducing formula, both through personal experiences and what mothers observed and heard within their communities.

Gender norms and perceived and embodied realities of breastfeeding emerged as interconnected sociocultural influences on feeding decisions within the household and broader community.

### Perceptions of breastfeeding promotion efforts - social transfer program

Participants were prompted to reflect on existing and proposed breastfeeding support initiatives, including social transfer programs. Mothers and fathers shared their perspectives on how such programs might be structured and whether social transfer support would be beneficial. Key stakeholders discussed implementation considerations, feasibility, and potential impacts on breastfeeding practices. These reflections provided insight into how such initiatives are perceived as mechanisms to address identified barriers and support breastfeeding.

#### Community perspectives

Mothers and fathers in the FGDs expressed broad support for social transfer programs, emphasizing the value of both financial and non-financial forms of support. Mothers noted that any form of support would be beneficial for both mother and child:

*Why can’t you theoretically be incentivized or rewarded for something you’re already doing for free … it will help you to improve the quality of life, both for the child and for yourself. Even if it is not a financial incentive, it can even be an educational incentive … It encourages your education, your child’s education and that will be a much better thing later on … (FGD 1, 32 years old)*

Mothers also emphasized that financial and food support would allow them to delay returning to work and spend more time breastfeeding:

*I think it would be more about food support, as you said, and financial support so that mothers don’t have to worry and have more time with their children and stay at home breastfeeding, it would have to be financial and food support so that they don’t have to work so early and leave their children so young. (FGD 2, 26 years old)*

A Bolsa Família recipient shared her views on the idea of a supplemental social transfer specifically targeted to breastfeeding mothers:

*I believe that for people who stay at home, who receive Bolsa Família, there should be a supplement to the amount. For example, I get R$750 because I have [my son], who is under four years old, and he gets an additional R$150. I think it would be good if there was an additional R$150 for mothers who need to breastfeed. It would help. (FGD 2, 27 years old)*

Fathers also reflected on the potential of a social transfer program to shift breastfeeding from an individual mother’s responsibility to a shared family commitment:

*I think this [program] encourages both the father and the mother to breastfeed, not just the mother. Not just the mother’s obligation … You bring the family together. In fact, we haven’t had, either from our family, or from the media, or from advertising, anything that would actually encourage the father to help with breastfeeding. (FGD 3, 51 years old)*

#### Stakeholder perspectives

Key stakeholders discussed social transfer programs primarily through the lens of implementation, feasibility, and structural impact. A MoH representative emphasized that a social transfer program could provide support similar to maternity leave, helping mothers and their families during the first six months postpartum:

*A woman who has a baby, she should have six months of some kind of benefit, whether it’s maternity leave or another benefit, but one that guarantees her six months. (KII 1, 43 years old)*

A healthcare worker highlighted that cash support would be particularly valuable for women in the informal sector:

*If she has this help, I think she’ll be more relaxed … Because these women who don’t have a job, they usually do informal jobs. Most of the time they don’t stay at home, so if she had extra income, she might be able to stay at home longer to breastfeed her child …. (KII 3, 72 years old)*

Another healthcare worker emphasized that cash support would be more beneficial than providing consumables, noting that rent and food are the primary financial concerns families face:

*From a perspective of: “to give you more support to stay at home, use this money to feed yourself, to pay your rent. There’s no need for you to go out to work and give up this time with your child”. I don’t think [consumables] would have the same impact, from the perspective I’m looking at it … it’s probably not the full cost of her living, but it could provide support for her. (KII 5, 45 years old)*

A MoSD representative raised concerns about conditional programs, highlighting both the additional pressure they place on mothers and the practical challenges of measuring breastfeeding:

*Zero sense for conditional programs, because you have all this background on the issue of breastfeeding. You have these barriers, which are cultural, structural and economic. So it’s going to end up being another penalty for this woman, who wasn’t able to breastfeed, and then the family is going to suffer … In the case of height and weight … height, you use a ruler. Weight, you can use a scale … How do you measure breastfeeding? How do you assess the greater or lesser degree of difficulty that a woman is facing … (KII 4, 43 years old)*

Several stakeholders noted broader public skepticism toward social transfer programs in Brazil, and some stakeholders and mothers were unaware of the existing Auxílio Nutriz program. A MoH representative reflected on the political barriers:

*We have to think of the financial impact as an investment, not an expense. But that’s not how it’s seen in Congress … investing in early childhood, in breastfeeding, is investing in the future. I think we have the political barriers, you can understand it as the polarization that happens here in our country, of saying that ‘people like to suck on the government teat’, that there will be people having more children because they want this benefit … These are words that are said, but there is no scientific proof that this is the case. (KII 1, 43 years old)*

Community members and stakeholders identified both potential benefits and key challenges related to the implementation and broader acceptance of social transfer programs to support breastfeeding.

## Discussion

Using insights from mothers, fathers, healthcare professionals, and ministry staff, this qualitative study explored perceptions and experiences that shaped barriers related to breastfeeding in the Butantã Jaguaré district of São Paulo, Brazil. Participants were also invited to reflect on existing and proposed breastfeeding social transfer programs as potential forms of structural and individual support, highlighting perceived benefits, challenges, and areas of improvement. Simultaneously examining community and stakeholder perspectives provides a multi-level understanding of how breastfeeding decisions are shaped by lived experience, social expectations, and institutional realities. While the majority of participants recognized the importance of breastfeeding, they described economic, health system, and sociocultural barriers that shape their ability to initiate and sustain breastfeeding. Although similar barriers have been documented in other settings, our findings are grounded in the Butantã Jaguaré district of São Paulo and should be interpreted within this specific urban context, as breastfeeding experiences and access to support may vary substantially across different areas of São Paulo.

**Fig. 1:**
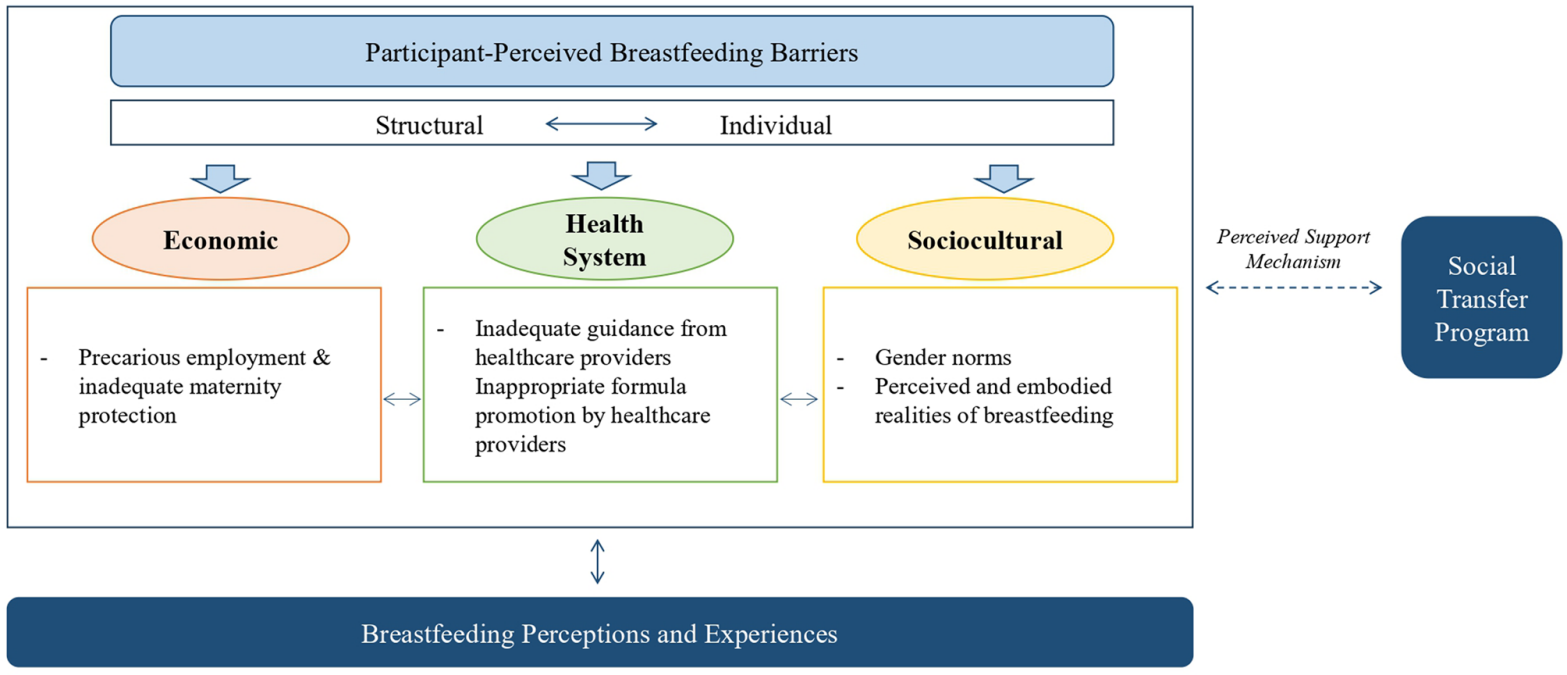
Conceptual framework illustrating participant-perceived barriers to breastfeeding, breastfeeding perceptions and experiences, and the potential role of a social transfer program

These findings can be understood through a social-determinants-of-health lens, where breastfeeding practices are shaped by broader structural conditions consistent with existing literature [26–29]. Economic constraints and limited maternity protection reflect well-documented barriers among women experiencing income insecurity or informal employment [27]. Health system barriers, including inadequate guidance and inappropriate formula promotion from healthcare providers, undermined mothers’ confidence and contributed to early cessation across institutional contexts [26–29]. Sociocultural barriers were equally prominent, with gender norms—including rigid division of household labor, partner authority over infant feeding decisions, and sexualization and appearance-related expectations—operating alongside perceived and embodied realities of breastfeeding shaped by stigmatization of breastfeeding, concerns about breast milk adequacy, and breastfeeding-related pain [26–30]. As illustrated in Fig. 1, these barriers operate at multiple interconnected levels and cannot be effectively addressed through individual-level interventions alone. When reflecting on social transfer programs as potential support mechanisms, participants commonly perceived that a well-structured intervention, such as Auxílio Nutriz, could help alleviate these barriers; for example, by reducing pressure to return to work prematurely, providing accessible breastfeeding guidance and support through healthcare professionals, and countering community-level stigmatization through family-centered education. Such programs should nonetheless be considered as part of a broader, multi-component approach that also includes improvements in employment protection, health system strengthening, and structural changes to address gender norms.

Reasons for early cessation of breastfeeding in the Butantã Jaguaré district of São Paulo varied widely between mothers but were consistent with patterns recounted in other studies. Participants emphasized that poor working conditions and financial instability were major barriers to breastfeeding. A systematic review by Kavle et al. reported financial pressures as a common factor for discontinuing breastfeeding [31]. Consistent with this, mothers in our study conveyed how insufficient maternity leave policies hindered them from breastfeeding exclusively for the recommended duration. A study in Mexico found that mothers working informal jobs are at a greater risk of breastfeeding cessation as they have more challenging working conditions, higher financial instability, and have little to no work protection [32]. In Kenya, one study found that 46.4% of mothers stopped exclusively breastfeeding before one month postpartum, primarily due to the need to return to work [33].

Participants often expressed the need for greater guidance from healthcare providers to improve breastfeeding continuation. Studies conducted in South Africa and Iran reported that a lack of support, education, and guidance from healthcare professionals were common barriers to breastfeeding [34, 35]. A report from Mexico highlighted how inadequate support during postpartum hospitalization discouraged mothers from initiating breastfeeding, as they were not taught proper breastfeeding techniques, often leading to difficulties and, consequently, to the use of infant formula [36]. Challenges such as pain and doubts about milk adequacy—which participants described as perceived barriers—have been associated in literature with inadequate postpartum breastfeeding support, suggesting that improved access to guidance in the postpartum period could help address these concerns [37, 38]. Information provided by health professionals to mothers was described as functioning either as a barrier or a facilitator to breastfeeding, depending on the quality and accessibility of guidance. Participants who indicated positive breastfeeding experiences frequently attributed them to supportive, well-informed healthcare providers.

The normalization and marketing of breast milk substitutes are widely documented in the literature as factors affecting breastfeeding prevalence [24, 39, 40]. Participants in our study similarly described encounters with formula promotion through healthcare professionals and online marketing. These experiences often intersected with moments of uncertainty about milk supply, bodily changes, limited support, or demanding work environments, influencing feeding decisions during vulnerable periods. Despite Brazil’s adoption of the WHO International Code of Marketing of Breast Milk Substitutes, which aims to regulate the promotion of infant formula and protect breastfeeding, prior research indicates that violations persist due to limited regulatory enforcement [12, 25, 41].

Sociocultural barriers shaped breastfeeding practices across multiple dimensions, encompassing gender norms and perceived and embodied realities of breastfeeding within the community. Consistent with findings from South Africa, participants emphasized that breastfeeding support extends beyond healthcare workers and should be shared by the broader community [34]. When the responsibility of breastfeeding is placed solely on the mother, it can create negative attitudes and contribute to early cessation, a pattern observed in our study. Traditional gender roles, where caregiving is primarily expected of mothers, were often perceived as contributing to feelings of overload and stress, which participants felt negatively impacted breastfeeding. This reflects broader evidence of unequal distribution of unpaid care work [42]. Breastfeeding is embedded within gendered expectations that position mothers as primary caregivers, shaping both emotional labor and feeding decisions [43, 44]. Notably, some participants described how partner authority directly overrode mothers’ feeding choices, suggesting that partner dynamics in this context extend beyond limited support to active constraint—a dimension that warrants greater attention in breastfeeding interventions targeting low-income urban communities. Participants further described experiencing pressure to breastfeed in environments where support and guidance within both the household and the community was limited. Studies have linked the sense of pressure to breastfeed with anxiety, stress, and depression, which are associated with early breastfeeding cessation [45].

Concerns about bodily appearance, including fears about breast sagging, or breast ptosis, were also identified as a barrier in our study, consistent with findings from other settings [46, 47]. Societal pressure to maintain physical appearance for partners has been shown to discourage some women from breastfeeding. Pressure from partners to keep up appearances can also lower breastfeeding efficacy [46–49]. This may be particularly pronounced in the Brazilian context, where body image norms are deeply embedded culturally and Brazil consistently ranks among the countries with the highest rates of cosmetic surgery worldwide, including breast augmentation [50, 51]. In this context, fears about bodily changes following childbirth may be amplified, even though research suggests these changes are primarily driven by pregnancy rather than breastfeeding independently [52, 53]. While research in southeastern Brazil suggests that negative body perceptions do not always alter feeding practices [30], a study in Tanzania found that aesthetic concerns did impact early breastfeeding cessation [54]. Similarly, participants in our study described concerns about bodily changes as a barrier to breastfeeding, noting that these concerns were particularly common among younger mothers in their communities.

Beyond gender norms, participants described perceived and embodied realities of breastfeeding that shaped feeding decisions and contributed to early cessation. Participants reported experiencing social pressure and judgment around breastfeeding, with cultural messaging creating stigma toward mothers who were unable or chose not to breastfeed—a pattern consistent with broader evidence linking perceived pressure to breastfeed with anxiety, stress, and depression associated with early cessation [45]. Similar to a study in Ghana, mothers in our study also described experiences of harassment and unwanted male attention when breastfeeding in public, highlighting how the absence of safe and supportive public spaces created additional barriers to maintaining EBF [55]. This reflects broader evidence that public breastfeeding remains a site of social tension, where mothers navigate competing pressures of cultural expectation and public discomfort.

Concerns about breast milk adequacy represented another widely held misconception among participants, consistent with findings from other low- and middle-income settings [33, 56–58]. A qualitative study conducted in Fortaleza, Brazil, found that the perception of having “weak” milk was associated with early weaning [57], and perceived breastmilk insufficiency has been identified as a primary driver of low EBF rates in Nairobi, Kenya [33]. In our study, uncertainty about milk supply frequently intersected with other challenges—including pain, social pressure, and limited support—compounding the decision to introduce formula. Breastfeeding was also commonly described as painful, particularly during initiation, with pain contributing to cessation especially when combined with other barriers [37, 38]. These perceived and embodied realities reflect how community-level beliefs, social norms, and bodily experiences shaped breastfeeding practices.

Social transfer programs have been shown to positively impact health outcomes, particularly in low- and middle-income countries [17]. Bolsa Família is a notable example that has improved child health outcomes [59]. Social transfer programs have been documented as a mechanism that may enhance maternal agency by increasing financial autonomy and reducing structural constraints, potentially allowing mothers more flexibility in feeding decisions [60].

A social transfer program targeting breastfeeding in Vientiane, Lao People’s Democratic Republic, has been associated with improvements in EBF rates [61]. Both conditional and unconditional transfers were found to improve breastfeeding outcomes [61]. However, the stakeholders interviewed in our study raised concerns about conditional programs. They noted that such conditions may add additional pressure on mothers, rather than providing support. They also emphasized that assessing breastfeeding practices is more complex than measuring other health outcomes—it requires attention not only to whether a mother is breastfeeding or not but also to the broader structural factors that influence her ability to do so. Another study in Mexico analyzed the feasibility of a conditional social transfer program for breastfeeding and concluded that, while such a program is feasible, long-term sustainability is crucial for improving outcomes [62]. The study further emphasized the need for broader systemic changes, including restructuring societal norms and workplace rights for mothers. Our study similarly found that stakeholders emphasized that lasting change is not possible without coordinated responses between different governmental sectors [63]. While participants perceived social transfer programs as a potentially supportive mechanism, further research is needed to evaluate their effectiveness in improving breastfeeding outcomes. Community members, including both mothers and fathers, focused on the practical household benefits of financial support. Mothers emphasized financial and food support to delay returning to work, while fathers reflected on the program’s potential to foster shared caregiving responsibilities.

Our findings suggest that potential opportunities to strengthen Auxílio Nutriz in the Butantã Jaguaré district would involve complementing the existing unconditional social transfer with three integrated components. First, structured breastfeeding education and lactation counselling for mothers, drawing on the participant-identified need for consistent, accessible, and supportive guidance from healthcare providers, including tackling misinformation about formula, concerns about milk adequacy, and strategies to manage breastfeeding-related pain. This is consistent with findings from Mexico, which identified that pairing a social transfer with educational resources and access to essential breastfeeding materials was critical for sustaining practices among mothers in informal employment [32]. Second, family-centered support that includes partners and extended family members, to foster shared caregiving responsibilities and encourage partner involvement in breastfeeding support. Third, community-based awareness initiatives, including addressing stigmatization of breastfeeding and challenging appearance-related and sexualization-based expectations that discourage mothers from breastfeeding—barriers that emerged consistently across participant groups and may undermine the benefits of a social transfer program alone. Together, these components reflect a multi-level approach that could contribute to addressing several of the barriers identified in this study and strengthen the impact of Auxílio Nutriz within its existing national framework.

### Limitations

Our study has several limitations. Although thematic saturation was achieved, the sample size remained relatively small, which may have influenced the range of perspectives captured. The FGDs were limited to mothers and fathers residing in a single district in São Paulo, which may limit the diversity of perspectives captured and the applicability of findings beyond this specific study setting. While efforts were made to reflect the demographic characteristics of the Butantã Jaguaré district, this cannot be confirmed due to the small sample size and the sampling approach used. Although the study included participants from lower socioeconomic backgrounds, disparities in representation were observed, with mothers and fathers of relatively higher socioeconomic status appearing to contribute more frequently. This may have limited the visibility of perspectives from lower-income participants.

Recruitment relied in part on snowball sampling, which may have introduced selection bias by drawing participants from overlapping social networks, potentially limiting the diversity of perspectives captured. Individuals who were more socially connected or more engaged within community networks may have been more likely to participate, while more marginalized or less connected individuals may be underrepresented. Social desirability bias was likely present in the fathers’ FGDs, where some responses appeared aligned with socially desirable views rather than personal opinions.

It is important to note that all KIIs were conducted with female participants who were identified as White, reflecting limited racial and demographic diversity among key informants. This homogeneity may have influenced the perspectives captured, particularly in relation to structural inequities and lived experiences of breastfeeding among more diverse populations in São Paulo.

## Conclusion

Our findings reveal gaps between breastfeeding intentions and practices, driven by interconnected economic, health system, and sociocultural barriers. Addressing these challenges requires policies and interventions that recognize the importance of structural changes to enable mothers to breastfeed. The study findings provide a foundation for strengthening existing initiatives such as Auxílio Nutriz by integrating unconditional social transfer with accessible lactation support, guidance, and education for mothers, their families, and communities to help address identified barriers and support sustained breastfeeding practices among vulnerable populations in Brazil.

Building on insights from this qualitative study, an ongoing randomized controlled trial is currently examining the effectiveness, feasibility, and acceptability of this integrated approach and its potential to influence breastfeeding outcomes [64]. Evidence generated from this evaluation could inform policy refinement and guide consideration of broader implementation through Auxílio Nutriz within Brazil’s national social welfare program, Bolsa Família.

## Electronic supplementary material

Below is the link to the electronic supplementary material.

Supplementary Material 1

## Data Availability

The data used and/or analysed during the current study are available from the corresponding author on reasonable request.

## Abbreviations

EBF: Exclusive Breastfeeding
FGD: Focus Group Discussion
KII: Key Informant Interviews
LAC: Latin America and the Caribbean
MoH: Ministry of Health
MoSD: Ministry of Social Development and Fight Against Hunger
WHO: World Health Organization
