# Supplementary material for: Barriers to breastfeeding and targeted breastfeeding social transfer programs in São Paulo, Brazil: a qualitative study
Source: Int Breastfeed J. 2026 Jun 19;21:75. doi: 10.1186/s13006-026-00865-2 (PMC13450255; doi:10.1186/s13006-026-00865-2)
Supplement: Supplementary file 1 [file 13006_2026_865_MOESM1_ESM.pdf]

Focus Group Discussion with Mothers

Introduction

I would like us to do a round of introductions to help us get to know each other a little better.

1. What is the best part of your week so far?
2. Can you tell me your name and age, and number of kids and their ages.

General Breastfeeding Questions

I'm going to start out by asking you some general questions about women in Brazil, such as their decisions and experiences about how to feed their babies. I will be asking questions later about your own thoughts and experiences, but for now we'll start more generally.

1. In your opinion, what do most women in your community think of breastfeeding?
   a. Possible prompts (Use these only if necessary): Beautiful and natural, or ugly and unpleasant? Convenient or a hassle? Difficult or easy?
   b. Why? What do you think shapes that?
      i. Probe: where a woman is from or where she currently lives, or her education level?
2. Can you tell me a little bit about how most pregnant women in your community initially <u>plan on feeding</u> their babies?
   a. Why do you think they choose that?
      i. Possible prompt: Who do you think has the biggest influence on whether a woman breastfeeds (e.g. medical providers, partner, spouse, friends, family, etc.)?
      ii. What are women told by midwives or birth attendants about how to feed their baby?
3. Do you think some women plan on not breastfeeding their child?
   a. Why do you think they choose that?
   b. Does breast augmentation affect ones decision to breastfeeding or not?
4. What are some concerns about infant feeding in general?
   a. With formula? Breastfeeding?
5. What are your thoughts and perceptions toward infant formula?
   a. Is formula commonly used to feed infants?
      i. What formula brand is commonly used?
      ii. Is formula easily accesible?
      iii. If you use or have used infant formula, how was it prepared?

    b. Do women in Brazil prefer using formula to feed their infants over breastfeeding? If so, why is that the case?

6. Now, can you tell me a little about how most women feed their babies once they are born?
    a. What do women typically do with colostrum (first milk after birth)?
    b. I understand some women give their baby water or other milks before their own milk comes in, can you tell me a little about that?
    c. Why do you think some women do not breastfeed?
    d. What about as they get older? How old is the baby once the women typically stops breastfeeding? Why do they stop?
    e. How do you think breastfeeding affects the health of the baby?
7. In your opinion, what do most men in your community think of breastfeeding?
    a. Why might some men want the woman to breastfeed?
    b. Why might they not want the woman to breastfeed?
8. Who would you ask if you needed advice about breastfeeding?
    a. What would happen if you did not follow their advice?
9. In some cultures, women may breastfed a child that is not theirs, what do you think of this practice?

Mother's experiences

10. In your opinion, what do you think are some of the major problems (barriers) women like you face when they want to exclusively breastfeed their children until 6 months of age? "Exclusive breastfeeding" means only giving the baby breast milk, and no water, no teas, no porridge.
    a. Have to go back to work. Probe: What they do with their children when they go back to work?
    b. Feeling exhausted. Probe: Is there anyone who can help with the chores?
    c. Not having enough milk. Probe: Why do they believe this?
    d. Thinking they have to give other foods. Probe: Who tells them to give other foods?
11. Do the new mothers have to go back to work soon after the baby is born? If so, how soon after the baby is born? How long is a mother away from her baby when she is at work?
    a. Who feeds the baby when the mother is away and what is the baby fed?

   b. Who makes these decisions?

12. If you have breastfed or are currently breastfeeding, do you feel this played a role in your relationship with your infant? With your husband?
    a. Do you feel the relationship with your baby and/or husband changed? How so?
    b. How did this change your intimate relationship with your husband?

Infant Feeding Experiences

Now I am wondering if you could tell me a little bit, about how <u>you</u> fed your infant(s). Remember there is no wrong answer here; we are just trying to understand what it was like for you.

13. When your most recent baby was born, did you ever breastfeed?
    a. Why or why not?
14. How did your partner/husband feel about breastfeeding?
15. Did you give you give your recent baby infant formula?
    a. Why or why not?
    b. If yes:
        i. what formula brand was used?
        ii. Is formula easily accesible? Is it costly?
        iii. How do you prepare the formula?
16. At what age did you start giving the child other liquids? What did you give them and why?
17. At what age did you start to give the child porridge or other easy to digest food? What did you give them?
18. At what age of the child did you stop breastfeeding altogether? Why did you stop breastfeeding?

Incentives for Breastfeeding

19. If you were to design a program that encouraged women to breastfeed once the baby was born – what would that look like?
20. One option is to give women a small gift (e.g. books or toys for her child), or transport vouchers while she is breastfeeding, what is your opinion of that?
    a. Do you think such programs make sense? Do you think they are fair?
    b. How do you think the partner/husband would feel about that?
    c. What kind of gifts would you prefer? Hygienic products, diapers, toys for the kids?
    d. Can you think of any reasons why this type of program might be problematic?

e. Are there any other things you could think of that might help a woman start breastfeeding or breastfeed longer?

Decision Making

21. In your household, who decides how to use cash and how is this decision made? Why? If you disagree on these decisions, how is the disagreement resolved?
22. Do you think it is appropriate for women to earn and manage household money? Please explain.
23. In your household, who decides when to send children to school? Are decisions different when they are made regarding boys' and girls' education? Why?

Closing

24. Do you have any further comments or thoughts on breastfeeding you would like to share with us, that I have not asked?

Once again thank you very much for your participation.

Focus Group Discussion with Fathers

Introduction

I would like us to do a round of introductions to help us get to know each other a little better.

1. What is the best part of your week so far?
2. Can you tell me your name and age, and number of kids and their ages.

General breastfeeding questions

3. Can you tell me a little bit about what you know about breastfeeding?
    a. Can be positive (e.g. health benefits etc.) or negative (makes it harder to leave the baby with others, woman's time is occupied with baby)
4. How do you feel about breastfeeding? (Actively support/encourage both positive and negative responses)
    a. What has shaped your feelings about breastfeeding?
    b. Has that changed since having a baby (e.g. did you feel differently before)?

Infant Feeding Experiences

5. When your wife/partner was pregnant, how was the decision about how to feed the baby made?
6. What or whom informed that decision?
    a. Prompt (if no one mentioned their role): Did you ever talk with your wife/partner about breastfeeding? What did you discuss?
    b. Did you support your partners decision to breastfeed or not breastfeed? If she went against your wishes, how did you feel?
7. Would you be willing to talk to your wife about breastfeeding if you hear something about it and felt she did not know it? If yes, probe.
8. What are your thoughts and perceptions toward infant formula?
    a. Is formula commonly used to feed infants?
        i. What formula brand is commonly used?
        ii. Is formula easily accesible to women in Brazil?
    b. Do women in Brazil prefer using formula to feed their infants over breastfeeding? If so, why is that the case?
9. How do you feel about breastfeeding?
    a. Do you feel your relationship with your child changed when your partner started breastfeeding?

    b. Do you feel your relationship with your partner changed when she started breastfeeding?

10. What are some of the challenges that mothers in your community have in breastfeeding?
11. In your community, do the new mothers have to go back to work soon after the baby is born? If so, how soon after the baby is born? How long is a mother away from her baby when she is at work?
    a. Probe: Who feeds the baby when the mother is away and what is the baby fed?

Incentives for Breastfeeding

12. If you were to design a program that encouraged women to breastfeed once the baby was born – what would that look like?
    a. Should the program focus more on women, or the men? Why?
13. One option is to give women a small gift (e.g. books or toys for her child) while she is breastfeeding, what is your opinion of that?
    b. Can you think of any reasons why this type of program might be problematic?
    c. Do you think such programs make sense? Do you think they are fair?
    d. How do you think men in your community would respond if women received gifts or transport vouchers for breastfeeding?
    e. What things (other than money) would be useful for a family to receive, to support/encourage breastfeeding?

Decision Making

14. In your opinion, is it acceptable for a woman to breastfeed around her husband? in public? Around family members? Around friends?
15. In your household, who decides how to use cash and how is this decision made? Why? If you disagree on these decisions how is the disagreement resolved?
16. Do you think it is appropriate for women to earn and manage household money? Please explain.
17. In your household, who decides when to send children to school? Are decisions different when they are made regarding boys' and girls' education? Why?

Closing

18. Do you have any further comments or thoughts on breastfeeding you would like to share with us, that I haven't asked?

Once again thank you very much for your participation.

Key Informant Interviews with Key Stakeholders

Introduction

1. Can you tell me a little bit about the context of breastfeeding in Brazil?
    a. What is the history of infant feeding practices here? (Possible prompts: how have women traditionally fed their infants, has that changed over time? Why?)
    b. ***(For those in policy)*** What efforts have there been on the policy level to support/encourage breastfeeding (i.e. baby friendly hospitals, educational awareness programs, etc.)?
    c. Is breastfeeding in Brazil culturally acceptable? Why or why not?
        i. Do women breastfeed in public? Around family members? Around friends?
2. What are the perceptions toward infant formula?
    a. Is formula commonly used to feed infants?
        i. What formula brand is commonly used?
        ii. Is formula easily accessible to women in Brazil?
    b. Do women in Brazil prefer using formula to feed their infants over breastfeeding? If so, why is that the case?
3. ***(For those in policy)*** Are there any policies in place in Brazil regarding infant formula?
    a. The WHO recommends that hospitals do not provide samples of infant formula to mothers. They recommend that babies receive breast milk. Do you think that the WHO's recommendation against infant formula is followed by hospitals?
        i. Do you think this affects mothers' food choices?
    b. If so, what happens if hospitals don't follow the policy?
        i. How often is the policy not followed?
4. If you were to design a program that encouraged women to breastfeed – what would that look like?
    a. Would you focus on policy, organizational change, or supporting the family directly?
        i. What type of program do you think would have the most immediate change? The most long-term, sustainable impact?

5. Now I'd like to ask you about programs to support breastfeeding that focuses on providing a social transfer (gift) to the mother for her breastfeeding efforts. Do you think this would encourage breastfeeding?
    a. Can you think of any reasons why this might be problematic?
    b. What types of reimbursement/gifts (i.e.books or toys, or services) might help a woman continue breastfeeding or breastfeed longer in order to meet the global recommendations?
6. What do you think about a large-scale social transfer program for breastfeeding that is similar to Bolsa Familia?
    a. What do you think about adding breastfeeding conditionality to the Bolsa Família program?
    b. Do you think this is important or could work?
    c. If so, what do you think is needed for this program to be successful?
    d. If so, what evidence do you think is needed to make it a nationally implemented government program?

Health professional or other expert opinions

7. What are the infant feeding practices you recommend? Why?
8. Who has a role in deciding how a woman feeds her infant?
    a. What role do you feel like you have in shaping infant feeding practices as a health professional or expert in the field?
    b. Do you support women's decision to breastfeed or not breastfeed?
9. What are the main barriers of breastfeeding for mothers?
10. Now I'd like to ask you a very open-ended question, and I want to encourage you to share whatever comes to mind - what would you like to see happen related to infant feeding in your community?

Closing

11. Do you have any further comments or thoughts on breastfeeding you would like to share with us, that I haven't asked?

Once again thank you very much for your participation.
